# Supplementary material for: The interactome of intact mitochondria by cross-linking mass spectrometry provides evidence for coexisting respiratory supercomplexes
Source: Mol Cell Proteomics. 2017 Dec 8;17(2):216–32. doi: 10.1074/mcp.RA117.000470 (PMC5795388; doi:10.1074/mcp.RA117.000470)
Supplement: Supplemental Data [file supp_RA117.000470_133922_0_supp_23978_fzffwf.zip › spectra_annotation/mito_spectra_annotation/23-1-2-1-1-1.pdf]

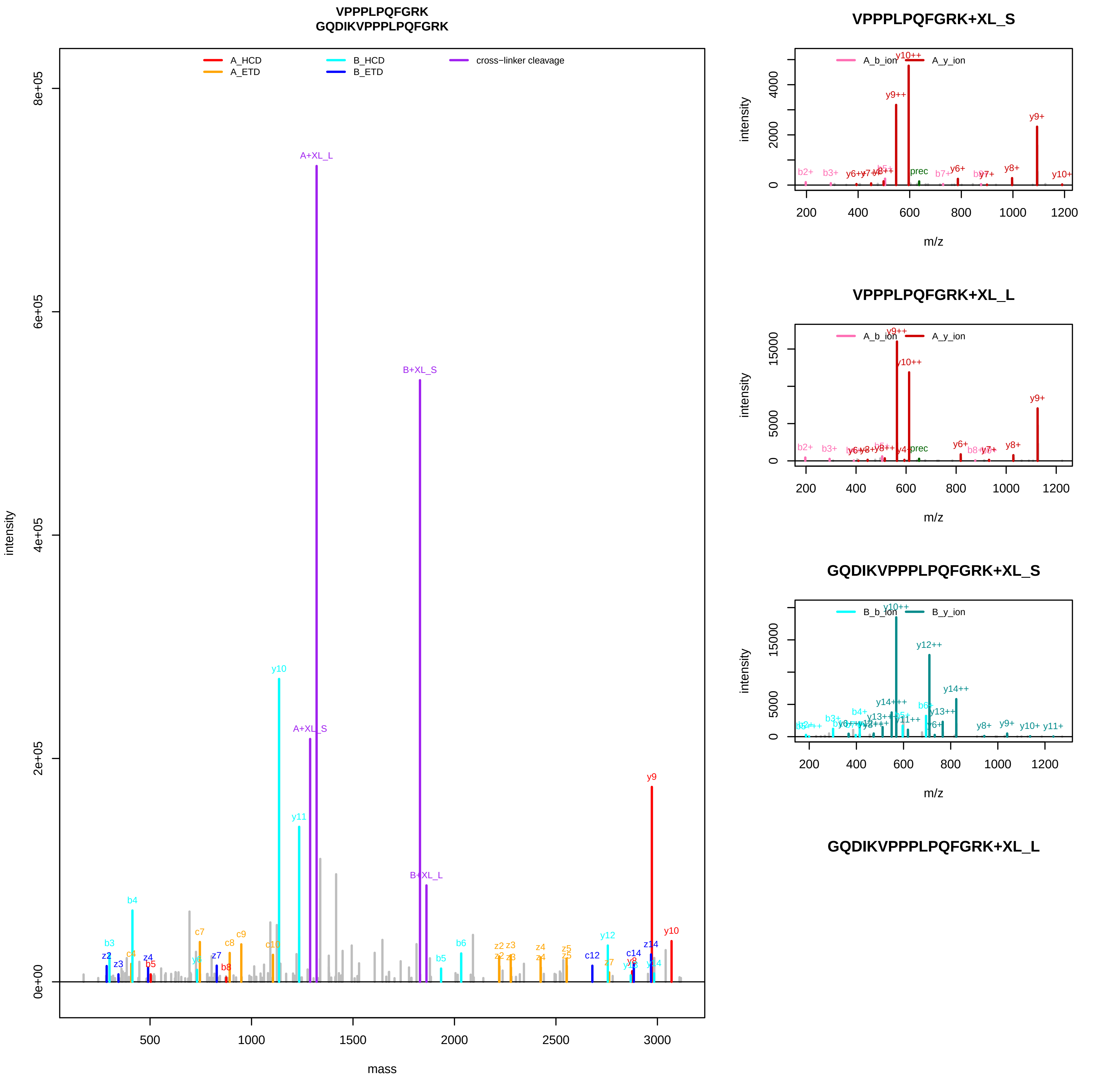
VPPPLPQFGRK
GQDIKVPPPLPQFGRK
A_HCD
A_ETD
B_HCD
B_ETD
cross−linker cleavage
intensity
mass
0e+00
2e+05
4e+05
6e+05
8e+05
500
1000
1500
2000
2500
3000
A+XL_L
B+XL_S
A+XL_S
B+XL_L
y10
y11
y9
y10
b3
b4
c4
z2
z3
z4
b5
c7
z7
b8
c8
c9
c10
b5
b6
z2
z3
z4
z5
c12
y12
z7
c14
z14
y14
VPPPLPQFGRK+XL_S
VPPPLPQFGRK+XL_L
GQDIKVPPPLPQFGRK+XL_S
GQDIKVPPPLPQFGRK+XL_L
A_b_ion
A_y_ion
B_b_ion
B_y_ion
intensity
m/z
y10++
y9++
y9+
y8+
y6+
y10+
b2+
b3+
b5+
b7+
prec
y12++
y14++
y14+++
y13++
y11++
b4+
b6+
y8+
y9+
y10+
y11+
200
400
600
800
1000
1200
